# The Effect of Relaxation Techniques on Anxiety, Fatigue and Sleep Quality of Parents of Children with Leukemia under Chemotherapy in South East Iran

**DOI:** 10.31557/APJCP.2019.20.10.2903

**Published:** 2019

**Authors:** Batool Pouraboli, Zeynab Poodineh, Younes Jahani

**Affiliations:** 1 *School of Nursing and Midwifery, Department of Pediatric and Neonatal Nursing, Tehran University of Medical Sciences, Tehran, *; 2 *Department of Community Health, Nursing and Midwifery School of Razi,*; 3 *Department of Biostatistic, Modeling in Health Research Center, Institute for Futures Studies in Health, Kerman University of Medical Sciences, Kerman, Iran.*

**Keywords:** Relaxation, anxiety, fatigue, sleep quality, leukemia, chemotherapy

## Abstract

**Introduction:**

Cancer can cause emotional stress in parents, which has a negative impact on the quality of their life. Also, anxiety and psychological stress have a negative effect on the health of parents, and fatigue causes a sense of weakness and reduces the capacity for mental and physical activity, and insomnia, as well as stress and inability to perform their occupational and social functions. This study aimed to determine the effect of relaxation techniques on anxiety, fatigue, and sleep quality of parents of children with leukemia under chemotherapy in South East Iran in 2015.

**Methods:**

This is a randomized controlled trial study. The study population included parents of children with leukemia undergoing chemotherapy who were admitted to a teaching hospital in South East Iran. One hundred twenty parents were randomly assigned to control and intervention groups, and the experimental group was provided with Benson relaxation technique. Data collection tool included a demographic questionnaire, state-trait anxiety inventory, Brief Fatigue Inventory, and sleep quality inventory. Data analysis was done by SPSS 16 and paired t-test, Wilcoxon, Mann-Whitney, regression, One - Way ANOVA and Pearson tests were performed, and p ≤ 0.05 was statistically significant.

**Results:**

The mean score of state anxiety in the intervention group was 60.86 ± 8.95 and 35.95 ± 4.61 before and after the intervention, respectively. The mean score of trait anxiety was 56.56 ± 4.75 and 34.45 ± 4.95. The mean score of the fatigue was 73.83 ± 14.63 and 43.71 ± 11. 06, and the mean score of the quality of sleep was 13.5 ± 6.05 and 5.7 ± 3.43 before and after the intervention respectively. There was a statistically significant difference among state-trait anxiety, fatigue, and sleep quality in intervention and control groups after the intervention. There was a statistically significant negative correlation between fatigue and age, but there was no statistically significant relationship among the mean fatigue, weight, the number of sons and daughters, education, occupation, gender, place of residence and income (p> 0.05). There was no statistically significant relationship among the quality of sleep of parents, education, gender, and place of residence, but there was a statistically significant relationship between state anxiety and education (p≤0.05).

**Conclusion:**

The results can predispose family-centered nursing care to support more the parents of children with cancer in the face of the stress of illness. Developing programs for training muscle relaxation techniques will improve family functioning and mental health.

## Introduction

Cancer is one of the main causes of mortality in the world (Papastavrou et al., 2009), the second cause of death in children in the third world countries (Kaatsch, 2010), the third main cause of mortality in Iran (Mousavi et al., 2010) and a new epidemic after heart disease (Papastavrou et al., 2009). Leukemia (34.1%) has been known as the most common childhood cancer in the world, and Iran (Kaatsch, 2010; Mousavi et al., 2010). Cancer causes a failure in the child’s life plans and family members, especially parents (Ozer et al., 2009) and chemotherapy in children with cancer, is a way to increase the length of life and survival of the child (Kyritsi et al., 2007). This increasing survival not only exposes the child to other health risks, including developmental-cognitive problems and changes in quality of life but also causes emotional stress in parents due to long-term exposure to these conditions, which negatively affects the quality of life of parents (Litzelman et al., 2011). Results show that the parents of children with leukemia are at risk of physical and emotional health related to the perceived dissatisfaction with the economic situation and the lack of adaptation (Khanjari et al., 2013). Factors that reduce the quality of life in the parents of children with leukemia are lack of adaptation with their children’s condition and anxiety, followed by fatigue and insufficient sleep (Tang et al., 2008).

Anxiety is one of the major problems of human society and psychological conditions (Bennett and Wells, 2010; Fiorentino, 2009) ranging from partial distress in daily activities to debilitating illness (Bennett and Wells, 2010). Stress, anxiety, and psychological stress in modern life, which are very complex and full of various problems and stresses, have a negative effect on the health of individuals (Mason et al., 2019). In parents of children with cancer, fatigue resulted from long-term treatment is one of the major problems that cause the highest level of fatigue. Insufficient sleep of mothers for better care of the child and the fulfillment of all wishes of the child is a symptom of her compassionate care and full involvement in the treatment indicating the highest level of parental self-sacrifice (Moridi et al., 2018).

The Keats study also suggests that parents prepare their cancerous children for a relatively long stay in a hospital and involve in their child’s treatment (Keats et al., 2018). Insomnia causes clinical stress and inability to perform occupational and social functions and has significant negative effects on the quality of life, and health status (Gebara et al., 2019). Controlling methods of fatigue, anxiety, and sleep quality include pharmacological and non-pharmacological methods (Georga et al., 2018). Non-pharmacological methods or complementary therapies include music therapy, yoga, hypnosis, energy therapy, massage therapy, acupressure, and relaxation (Ghafari et al., 2008).

The use of these non-pharmacological interventions is expanding among nurses (Zargarani et al., 2018). Muscle relaxation by balancing the posterior and anterior hypothalamus decreases the activity of the sympathetic nervous system and secretion of catecholamines reduces muscle tension, regulates respiration, reduces heart rate and muscle spasm, reduces physiological adverse effects, anxiety, and fatigue (Singh, 2018). 

Modanloo et al., (2014) studied family functioning in parents of children with cancer to determine the family functioning in the parents of children with cancer. They showed that paying attention to improving the functioning of families with a cancer child and supporting them in the problem-solving dimension were the main priorities of health and treatment (Modanloo et al.,2019).

Rajni (2018), studied “*Coping Strategies for Parents of Children with Cancer*” The results showed that the design and implementation of educational – counseling interventions for supporting caretakers of this group of children could improve their quality of life.

Rajni et al.,(2018) studied the concept of stress adjustment in caregivers of children with cancer, and the results showed that nursing interventions had to be done to reduce the stress of caregivers, and to train the use of coping skills of caregivers (parents and close relatives) of children with cancer.

Since, nursing care, child and family health are family-centered, family members of children with leukemia, especially their parents, are exposed to stress and anxiety caused by illness and long-term treatment of their child, the question is whether the intervention techniques such as muscle relaxation can be effective in reducing the stress and improving the quality of life of the parents of children? 

*Objectives*

This study aimed to determine the effect of muscle relaxation technique on anxiety, fatigue, and quality of sleep in parents of children with leukemia under chemotherapy in Zahedan Teaching Hospitals in 2015.

## Material and Methods

*Study design and setting*

The present study is a semi-experimental and randomized controlled intervention. The research setting was Teaching Hospitals in Zahedan such as Ali ibn Abi Taleb. 

*Study sample size*

50 samples at a confidence level of 95% and a test power of 80% were selected, but regarding drops out, 60 samples were evaluated in each group according to the statistic consultant.

**Table 1:** The Effect of Muscle Relaxation Technique on Fatigue, State Anxiety and Sleep Quality before and after Intervention among Parents of Children with Leukemia Treated with Chemotherapy in Educational Hospitals of Zahedan in 2015

Dimensions	Group	Mean ±SD		Test statistics, P value
		Before	After	
Fatigue	Intervention	73.38± 14.63	43.71±11.06	T= 46.41, P= 0.0001
state anxiety	Control	57.56±4.81	53.26±3.60	T= 12.13, P= 0.110
Sleep quality	Intervention	13.50 ± 6.05	5.7±3.43	T=19.11, P= 0.0001

**Table 2:** The Effect of Muscle Relaxation Tchnique on Fatigue, State-Trait Anxiety and Sleep Quality before and after Intervention among Parents of Children with Leukemia Treated with Chemotherapy in Educational Hospitals of Zahedan in 2015

Variable	Group	Mean ± SD		Test statistics, P value
		Before	After	
Fatigue	Control	62.46 ± 20.19	54.36 ± 10.43	Z= - 4.75, P= 0.053
state anxiety	Intervention	60.86 ± 8.95	35.95 ± 4.61	Z = - 6.74, P= 0.0001
trait anxiety	Intervention	56.56 ± 4.75	34.45 ± 4.95	Z= - 6.74, P= 0.0001
trait anxiety	Control	56.35 ± 4.46	53.13 ± 3.05	Z= - 5.91, P=0.112
Sleep quality	Control	13.73 ± 6.41	13.25 ± 3.35	Z= - 6.39, P= 0.101

**Table 3:** The Effect of Muscle Relaxation Technique on Fatigue, State-Trait Anxiety and Sleep Quality between Two Groups of the Intervention and Control in the Parents of Children with Leukemia Treated with Chemotherapy in Educational Hospitals of Zahedan in 2015

Variable	Group	*Diff mean ± SD	Test statistics, *P-value*
Fatigue	Intervention	30.11 ± 5.02	Z= - 9.24
	Control	8.10 ± 10.64	P= 0.0001
state anxiety	Intervention	24.91 ± 5.03	Z= - 9.44
	Control	11.00 ± 4.30	P= 0.0001
trait anxiety	Intervention	22.11 ± 4.08	Z= - 9.39
	Control	8.00 ± 3.21	P= 0.0001
Sleep quality	Intervention	7.80 ± 3.16	Z= - 9.25
	Control	- 6.51 ± 3.55	P= 0.0001

* Difference (the mean before and after intervention)

*Intervention*

After selecting 120 people, they were assigned to experimental and control groups (60 people in each group) by random block allocation using software R version 3.01.2. This study, with a potential of 80%, can detect the difference in the anxiety score between the two groups in at least scores 3 and higher. In this study, the samples were selected in 4 blocks (so that the group A is intervention and group B is a control) and divided into two groups of intervention (60 subjects) and control (60 subjects). Intervention in this study was muscle relaxation, which is a type of respiratory relaxation, is very easy to use for most parents, and it is associated with decreased sympathetic nervous system activity. The educational content of the sessions included questions and answers about the benefits of relaxation and practical presentation of the relaxation technique that was carried out twice a week in the presence of the researcher. After explaining how the technique was performed, research samples were asked to perform practices in the presence of the researcher to ensure that they were doing practices accurately, and they were asked to perform these practices twice every day for 20 minutes. The samples were contacted by telephone calls to follow the relaxation technique. The educational pamphlet with the CD was also provided for the research subjects and the state-trait anxiety inventory, FBI and sleep quality inventory were completed by the intervention group before the intervention and four weeks after the relaxation exercises.

*Measurements*

The data gathering method was a questionnaire which was completed in the form of an interview by subjects who were not literate. The demographic characteristics of the research units included: age, weight, sex, marital status and blood type, number of children, occupation, and level of education, income, and place of residence. Spielberger state-trait Anxiety inventory, an instrument to measure anxiety, has been designed and published by Spielberger. The test consists of forty phrases that are expressed in two state and trait anxieties, each containing twenty items based on the 4-point Likert scale. The Brief Fatigue Inventory (BFI), which consists of 10 items, measures the current fatigue, the usual fatigue in the last 24 hours, the highest rate of patients’ fatigue in the past 24 hours, the effect of past 24-hour fatigue on overall activity, mood, walking ability, association with other people and the living enjoyment. The Pittsburgh Sleep Quality Inventory (PSQI), which includes nine questions, measures sleep quality.

*Statistical Analysis*

Data were collected by SPSS software. Data description was done using central, dispersion indicators, frequency and percentage. For the difference between the scores of fatigue and sleep quality before and after intervention in the intervention group and trait anxiety in the control group to be compared, paired samples t-test was used due to the normalization of the data. Wilcoxon test was used to compare the score difference of state-trait anxiety before and after intervention in the intervention group and fatigue, trait anxiety, and sleep quality in the control group. Mann-Whitney test was used to determine the effect of muscle relaxation on anxiety, fatigue, and sleep quality between intervention and control groups. Also, parametric tests such as t-test, Pearson correlation and One-Way ANOVA were used to correlate each dimension of fatigue, state-trait anxiety, and sleep quality with demographic variables, considering the fact that the mean difference scores of fatigue, state-trait anxiety, and sleep quality and quantitative demographic variables were in the normal distribution before and after intervention.

*Ethical considerations*

1. Obtaining the informed consent of the participants and keeping their privacy

2. Ensuring the participants’ information confidentiality 

3. Explaining to the research units about the purpose and the duration of the project

4. Explaining the possibility of withdrawing the study at any stage in case of unwillingness to continue cooperation

5-Providing the results of the research for the subjects and staff of the ward and hospital

6- Providing an emergency call number for more information 

This study with the code of ethics IR.KMU.REC.1394.599 has been registered in Kerman University of Medical Sciences and Health Services.

## Results

The mean age of parents of children with leukemia in the intervention group was 36.9 with the standard deviation of 10.9, and it was 38.06 in the control group with a standard deviation of 12.62. 53.4% of the parents had one or two daughters, and 33.3% of them had three sons. The majority of participants (40%) were illiterate, 30% did not have a diploma, and 30% had a diploma. 43.3% were living in the city, and 7.56% were living in the village, and the majority of parents (93.3%) reported income under one million toman. Also, 43.3% of these parents were housewives, 13.3% were employed, and 38.3% of them had self-employed jobs.

Based on paired samples t-test, there was a statistically significant difference in each dimension of fatigue and sleep quality in the intervention group before and after the intervention (p = 0.0001) (Table 1).

According to Wilcoxon test, there was a statistically significant difference between the two groups before and after the intervention in each of the variables of fatigue, state-trait anxiety, and sleep quality of the parents of children with leukemia under chemotherapy (p= 0.0001) (Table 2).

According to the Mann-Whitney test, there is a significant difference in the dimensions of fatigue, state-trait anxiety, and sleep quality between the intervention and control groups in the parents of children with leukemia under chemotherapy (Table 3).

Based on the Pearson correlation test, there was a statistically significant negative correlation between fatigue and age (p = 0.046) and the older the age, the lower the fatigue. Also, sleep quality had a negative and significant relationship with the number of sons (p = 0.039), and the higher the number of sons, the lower the sleep quality. Also, based on the ANOVA test, there was a statistically significant relationship between education and state anxiety (p = 0.048), and there was a significant relationship between occupation, and state-trait anxiety. There was no relationship among fatigue, state-trait anxiety, sleep quality, and other demographic variables.

## Discussion

Cancer is a disease that has a severe impact on the patient, as well as the family and parents, which leads to a change in the parent’s lifestyle. In the Modanloo et al., (2019) study; the parents’ primary response to the diagnosis of leukemia was the fear and anxiety throughout their life because they considered cancer as “*an imminent death*” In this regard, the results of a study entitled parents burden of care for children with cancer also found that parents’ burden of care was more than moderate. In a study done by Kalhor et al., (2014) with the aim of explaining the experience of parents of a child with leukemia, parents came to a dead end in the life which was identified as one of the main themes of research. Therefore, the stress imposed on the parents is considered stronger and heavier due to strong family dependency. 

The results of this study showed that the mean of anxiety and stress in the intervention group decreased compared to before the intervention. The results of other studies have shown that the level of anxiety and stress in people can be greatly reduced by relaxation (Lazaridou and Edwards, 2019). The results of another study show that those who attend stress coping classes and who are living with family members have less stress and anxiety than those who do not attend the classes, are living alone and without family members. (McDonough et al., 2019). The results of this study showed that the use of a relaxation method could reduce the anxiety of parents of children with cancer in the intervention group. Using relaxation methods, one can establish an effective relationship with parents in the process of treating children with cancer, which may lead to increased parental satisfaction. According to the results of the study, it can be recommended that relaxation-based treatment of chronic diseases such as cancer should be preferred by both child and parents since it has no complication as well as no additional cost to the treatment system and patient. 

In the fatigue dimension, statistical tests showed that there was a statistically significant difference between the intervention and control groups after the intervention in the parents of children with leukemia under chemotherapy. In the study results of Harper et al., (2019), fatigue has been considered as another major problem for parents and children with cancer due to the long course of treatment. In another study, there is a high level of fatigue both in parents and in children with leukemia (Wu et al., 2019). In a study by Yurtekuran et al., (2007), an exercise program was done for hemodialysis patients. The results showed that complementary treatment methods produced a significant difference in the mean score of fatigue between the intervention and control groups and complementary treatments could reduce patients’ fatigue. Tayyebi et al., (2011) conducted a study to investigate the effect of yoga (with particular attention to relaxation in the body) on the hemodialysis patients’ fatigue. The final result of their study showed that yoga could reduce fatigue. The present study showed that muscle relaxation technique decreased fatigue in the intervention group compared to the control group. This result can indicate the effect of relaxation on reducing the level of fatigue. According to the results of the study and the ability to implement a good and easy technique of muscle relaxation as a complementary therapeutic method, it can be said that a relaxation technique can reduce fatigue which is one of the most debilitating symptoms amongst parents of children with leukemia.

Regarding sleep quality, pre-intervention mean was maximum in intervention and control groups, which showed poor sleep quality, and the post-intervention mean decreased significantly. Based on the results of the tests, there was a significant difference between the intervention and control groups after intervention in sleep quality and the parents of children with leukemia under chemotherapy. In Valizadeh et al., (2014) study, after a definite diagnosis of leukemia in children, mothers decided to stay with their children at the hospital during the night, and the fathers also came to visit children after their work hours. In the study of Ames et al., (2011) in Canada, parents also had full involvement in caring for their child in the intensive care unit, blood unit, and oncology of children. In a study by Lam et al., (2007), the parents also were ready for a relatively long stay in the hospital and had a strong desire to participate. Other studies have shown that complementary treatments such as energy conservation strategies are effective in reducing patients’ fatigue. According to these studies, it has been shown that a one-to-twenty-minute session of relaxation saves and stores energy in person as a one-hour sleep (Shariati et al., 2010). A study by Akbarzadeh et al., (2014) aimed at the effect of Benson relaxation method on the sleep quality of patients with chronic heart disease showed that relaxation of Benson as one of the components of cognitive-behavioral therapy had a significant effect on the quality of sleep in patients with coronary heart disease. The present study showed that progressive muscle relaxation significantly improved the sleep quality of parents of children with cancer, while the higher difference in other dimensions was observed in the intervention and control groups, which can be considered as a proper method to improve their sleep quality and quality of life. Staying in the hospital for child care will affect the quality of sleep because it will disturb the normal conditions of one’s life. There are several factors involved in it, hospitalization is in the first place, and other factors are anxiety and stress, being away from the family, as well as the change of the place of sleep and its inappropriateness, the presence of triggers such as noise and light..

In Conclusion, in general, these results can predispose family-centered nursing care to support more parents of children with cancer in the face of illness-based stress. With proper educational interventions such as family therapy and education of parents in groups and the dynamic process of the group, the family function can be improved, and steps can be taken to empower families. Improving the family’s function will enhance the mental health of the family and society, and improve the quality of life of all the family members and the ill child. It is necessary to develop cohesive plans and programs for parents of children with cancer to attend classes and to teach muscle relaxation techniques besides other parents and to understand the circumstances of each other.

*Limitations*

Some research units were uneducated, and interviewing was used to complete the questionnaires. The research units might refrain from expressing the truth because of fear of being identified. We tried to control this limitation and explained to them about the questionnaires’ anonymity, the purpose of the research, and the use of information only for the research purpose and ensured them about information confidentiality.
